# Examining constipation assessment and management of patients with advanced cancer receiving specialist palliative care: a multi-site retrospective case note review of clinical practice

**DOI:** 10.1186/s12904-019-0436-3

**Published:** 2019-07-15

**Authors:** Sonja McIlfatrick, Deborah H. L. Muldrew, Esther Beck, Emma Carduff, Mike Clarke, Anne Finucane, Lisa Graham-Wisener, Phil Larkin, Noleen K. McCorry, Paul Slater, Felicity Hasson

**Affiliations:** 1 Institute of Nursing and Health Research, Ulster University, Shore Road, Newtownabbey, Co Antrim BT37 0QB UK; 2 Marie Curie Hospice, Balornock Rd, Glasgow, G21 3US UK; 3 Queen’s University Belfast, University Rd, Belfast, BT7 1NN UK; 4 Marie Curie Hospice, Frogston Road West, Edinburgh, EH10 7DR UK; 5 Université de Lausanne, Lausanne, Switzerland

**Keywords:** Constipation, Symptom management, Palliative care, Hospice: specialist palliative care, Chart review

## Abstract

**Background:**

Constipation is a common symptom for patients receiving palliative care. Whilst international clinical guidelines are available on the clinical management of constipation for people with advanced cancer receiving specialist palliative care (SPC), the extent to which the guidelines are implemented in practice is unclear. This study aimed to examine clinical practices for the assessment and management of constipation for patients with advanced cancer within inpatient SPC settings.

**Methods:**

A multi-site retrospective case-note review was conducted, consisting of 150 patient case-notes from three inpatient SPC units across the United Kingdom between August 2016 and May 2017. The variables selected for review were determined by the recommendations within the clinical guidelines. Descriptive statistics, cross tabulation, chi square, and bivariate correlations were used to examine clinical practices compared to policy guidelines for the assessment and management of constipation. Reporting was structured by the STROBE checklist for observational research (Additional File 2).

**Results:**

A comprehensive assessment, including a full history and performing a physical exam, was recorded for 109 patients (73%), however, no standardised documentation was utilised. Assessment was nurse led, with variable involvement across sites of other members of the multidisciplinary team (MDT). Education on prevention was documented in 30 (20%) case-notes, and 53% recorded evidence of non-pharmacological intervention. Age, gender, and reason for admission did not impact on the likelihood of receiving a comprehensive assessment, education, or non-pharmacological intervention, however, significant differences were evident between sites. Pharmacological management was well developed and aligned to the guidelines however, 33% of patient case-notes recorded no information on the titration of laxatives. Twelve percent of patients experienced partial or complete bowel obstruction, and management strategies were variable.

**Conclusions:**

Constipation management is driven by a pharmacological approach, with little evidence of the implementation of preventative and non-pharmacological strategies. The nurse plays a key coordinating role in assessment; however, involvement and roles of the wider MDT varies. Accurate recording of care is essential when examining clinical practice and identifying areas for improvement. Further education is needed to equip HCPs with the knowledge and skills to ensure consistency in assessment and implementation of appropriate non-pharmacological/ preventative strategies.

**Electronic supplementary material:**

The online version of this article (10.1186/s12904-019-0436-3) contains supplementary material, which is available to authorized users.

## Background

Constipation is identified by unsatisfactory defecation due to infrequent stools, difficulty passing stools, or the sensation of incomplete emptying [1]. This contributes to considerable physical and psychological suffering for patients and their families [2]. It is one of the most frequent gastrointestinal complications encountered in clinical practice in Western societies [3], with prevalence rates increasing in older adults across community [4] nursing homes [5], and specialist palliative care [6] settings. Whilst constipation affects approximately 40% of patients receiving palliative care [7], this figure increases in specialist palliative care (SPC) to greater than 66% [6]. Mercadante [6] assessed the change in prevalence of constipation in patients with advanced cancer after one week of receiving support from SPC. Patients with low bowel function (bowel function index ≤28) experienced worsening of their symptoms, and patients with normal bowel function at initial assessment saw a worsening in their condition due to lack of prevention or subsequent under-treatment. A systematic review of constipation in SPC settings identified challenges for the assessment and management of this symptom, including a lack of standardised assessment, an overemphasis on pharmacological intervention, and lack of preventative and non-pharmacological intervention [8]. Fragmented approaches to constipation management were highlighted in a European Report on the burden of constipation in an ageing population [9], and similar challenges have been identified in previous hospice-based studies, identifying a need to improve the overall clinical practices of assessment and management of this symptom [10].

To guide clinical practice, a review of guidelines identified twenty-two generic clinical guidelines for constipation across North America, Europe, and Asia [11]. It was found, however, there was only one guideline developed specifically for palliative care, developed according to the principles of the ADAPTE^1^ process [12] (Additional file 1). These guidelines provide a useful starting point for evidence-based practice as their recommendations are well supported by the research literature. Such recommendations include: the need for a comprehensive assessment using the Rome III criteria [13]; a complete documentation of medical history; and the use of physical examination as part of the comprehensive assessment process [14–17]. In order to ensure effective management of constipation, it is recommended that equal attention is paid to optimised toileting, lifestyle modifications, and adjustment of activity levels, alongside pharmacological management [18]. Very recent clinical guidelines have been developed by the European Society for Medical Oncology for the diagnosis, assessment, and management of constipation [19], and similar key recommendations are made compared to the previous guidelines. This includes the need for a comprehensive assessment, and a balance of strategies for prevention and self-care and prescribed laxative therapy. It is noteworthy that despite a systematic review of clinical trials highlighting no evidence to support the use of one laxative over another [20], previous research in both the UK and USA identified preferences for specific laxatives, such as Senna and Sodium Docusate [10, 21]. It is also noted that clinical record keeping is an integral component in the delivery of quality healthcare [22, 23], and essential to ensure high quality care. Whilst key areas have been identified by the guidelines and supported by the literature, questions exist for these specific guidelines but also more generally across oncology guideline implementation around the extent of their implementation, HCP compliance to the guidelines across patients, and impact in clinical practice [24]. There is a dearth of real-world, patient-level, clinical evidence regarding the management of constipation for people with advanced cancer in SPC settings.

## Methods

### Aim

To examine the clinical practice for the assessment and management of constipation for patients with advanced cancer within SPC settings.

### Design

A descriptive, retrospective, observational study was conducted. Multidisciplinary case-notes of patients (*n* = 150) were reviewed in three SPC inpatient units. These notes were reviewed using a process guided by Gilbert’s [25] eight criteria, which includes abstractor training, explicit case selection, clear definition of variables, standardised abstraction forms, meetings with abstractors to resolve disputes, monitoring performance of abstractors, blinding of abstractors, and testing for inter-rater agreement. These criteria have been previously applied within palliative care research focusing on end of life care provision, medication use, and hospitalization [26] and were therefore deemed appropriate for use in the current study. Reporting was structured by the STROBE checklist for observational research to provide a rigorous structure for reporting and to ensure sufficient information is presented to allow for replication [27] (Additional file 2).

### Setting

In the United Kingdom (UK) in 2014/2015, at least 36,000 people used SPC inpatient services; 80 % of these patients had a diagnosis of cancer, and typically were admitted as part of a planned series of short stays between one and four days [28]. Data were collected from a convenience sample of three SPC inpatient units associated with one hospice organisation. The sites included represented three distinct regions of the UK, had previously established collaborative links through involvement in other research projects, and had expressed an interest in this particular topic. The units comprised on average 22 beds, and admitted on average 329 patients each year. Patients admitted from August 2016 to May 2017 were included in the review.

### Participants

On average, 987 patients are admitted to the three hospices annually, of which approximately two thirds may experience constipation. In conjunction with the research leads at each clinical site, 150 patient case notes were identified as an appropriate sample (50 per clinical site) within a ten-month period to enable a suitable comprehensive picture within the timeframe and available resources. Case-notes were reviewed chronologically from the beginning of the selected time-period until 150 had been identified which met the criteria:

- Adult patients with advanced cancer;
- Aged over 18 years;
- Admitted to the hospice in a specified period (if a patient was readmitted, data were only extracted for their first admission in this time period); and
- Constipation was noted as a problem in their multidisciplinary records as per the Rome III criteria.

Patient records were reviewed and data extracted using a standardised data extraction form, adapted from clinical guidelines for the management of constipation [18], and in consultation with experts from the clinical sites (See Additional file 1).

### Data collection

Six health care professionals (HCPs) (five nurses and one physiotherapist) with an interest in this clinical area and working in the hospice volunteered as data abstractors. A training session was held prior to data abstraction to clarify inclusion criteria and practical considerations when undertaking the review. Data abstractors accessed the patient’s clinical notes, either through an online system or paper records, depending on accessibility at the site. After data abstraction from five patient case-notes per site, a follow up meeting was held to discuss any problems and ensure consistency in data recording.

### Variables

Key variables for the review included assessment, prevention, pharmacological and non-pharmacological management strategies, opioid induced constipation (OIC), and intestinal obstruction. Additional data included patient demographics, medical history, and reason for referral. The variables selected for review were determined by the recommendations within the clinical guidelines [18]. The use of a standardised form, multiple training sessions, and ongoing discussions between the research team and data abstractors increased confidence in the standardised completion of the forms.

### Bias

To minimise the risk of selection bias, consecutive, chronological cases were selected to prevent data abstractors picking cases reflective of better practice. Measurement bias was managed by selecting data abstractors who were separate to the research team and had no prior investment in the project. To prevent the risk of publication bias, all findings from the review are presented, including ones which may demonstrate poor documentation or poor clinical practice.

### Data analysis

Data were entered into IBM SPSS Statistics v23 and descriptive analysis undertaken. Variables were compared across sites for similarities and differences. Cross tabulation and chi squared tests were used to examine differences across variables. Bivariate correlation examined relationships between continuous variables. If “not recorded” was noted for a question in more than 15% of clinical case-notes, it has been discussed in the results as this is a commonly cited maximum for acceptable missing data [29, 30].

### Ethics

The study protocol, including case note review, was approved by the Office for Research Ethics Committees Northern Ireland (ORECNI) (REC Reference 16/WM/0352). To access case notes, each hospice site’s governance committee granted approval. Patient data were anonymised before being transferred to the research team, ensuring no patient could be identified within the data.

## Results

### Demographic profile

One hundred and fifty case-notes were reviewed (see Table 1). The mean age of patients was 69 years (SD = 12.09). Over half of patients were female (*n* = 85; 56.7%), and the main reason for admission was noted as symptom control (*n* = 71; 47.3%). A third of patients were recorded as living independently prior to admission (*n* = 50; 33.3%) with family support (*n* = 132; 88.0%). The most frequent primary cancer sites were upper gastrointestinal (*n* = 35; 23.4%), lung (*n* = 29; 19.3%), and urological (*n* = 20; 13.3%). The mean length of stay in the SPC inpatient unit was 18.83 days. Patients were prescribed on average 1.25 laxatives (SD = 1.10) and 1.45 opioids (SD = 1.03). Medicines which acted on the gastrointestinal system were prescribed most frequently (mean = 2.31; SD = 1.40), followed by central nervous system drugs (mean = 2.26; SD = 1.91) and drugs specifically for pain (mean = 2.16; SD = 1.52).

**Table 1:** Demographic profile of patients

Characteristics		N (%)
Location	Northern Ireland (Belfast)	50 (33.3)
	Scotland (Edinburgh)	50 (33.3)
	England (West Midlands)	50 (33.3)
Gender	Male	65 (43.3)
	Female	85 (56.7)
Reason Admitted	Symptom Control (Physical or Psychological)	82 (54.7)
	End of Life Care	65 (43.3)
	Other	1 (.7)
	Missing	2 (1.3)
Referral Source	Specialist Palliative Care Team	65 (43.3)
	Hospital (including outpatients)	50 (33.3)
	Community Referral (including General Practitioner and Nursing home)	29 (19.3)
	Not Recorded	2(1.3)
	Missing	4 (2.7)
Primary Cancer Site	Upper GI	35 (23.4)
	Lung	29 (19.3)
	Urological	20 (13.3)
	Breast	11 (7.3)
	Colorectal	11 (7.3)
	Gynaecological	11 (7.3)
	Haematological	9 (6.0)
	CNS	8 (5.3)
	Head and Neck	7 (4.7)
	Skin	3 (2.0)
	Bone	2 (1.4)
	Other	4 (2.7)
Mobility	Independent	50 (33.3)
	Assistance of One	34 (22.7)
	Assistance of Two	31 (20.7)
	Bed Bound	32 (21.3)
	Missing	3 (2.0)
Family Support	Yes	132 (88.0)
	No	11 (7.3)
	Missing	7 (4.7)

### Main findings

The results from the case note review are noted and summarised against the key areas as outlined within the clinical guideline recommendations (see Table 2). The results from each of these respective areas are then outlined.

**Table 2:** Case note comparison to guidelines

	Case-note review question	All sites n(%) yes
Clinical Guideline: Assessment		
1.1 A thorough history and physical examination are recommended as essential components of the assessment process.	Was a comprehensive assessment carried out?	109 (73)
Constipation assessment scales are **not** recommended for routine use.	Was an assessment tool used?	144 (96)
1.3 A digital rectal examination (DRE) is required to exclude faecal impaction if it has been more than 3 days since the last bowel movement or if the patient complains of incomplete evacuation	^a^DRE performed when it’s been 3 or more days since last evacuation	25 (17)
	^a^DRE performed when the patient complains of incomplete evacuation	2 (1)
1.5 A plain film of the abdomen (PFA) is not recommended for routine evaluation but may be useful in combination with history and examination in certain patients	Was a PFA performed?	5 (3)
Clinical Guideline: Education		
2.1 Education on the importance of non-drug measures is essential to enable patients and caregivers to take an active role in constipation prevention.	^a^Was education on non-drug measures recorded?	30 (20)
Clinical guidelines: Management		
3.1 Attention should be paid to the provision of optimised toileting while ensuring adequate privacy and dignity.	^a^Was there evidence of consideration of optimised toileting?	28 (19)
	^a^Was there evidence of consideration of privacy?	52 (35)
3.2 Consideration should be given to lifestyle modification (adjustment of diet and activity levels within a patient’s limitations).	^a^Was there evidence of consideration of diet and fluids?	55 (37)
	^a^Was there evidence of consideration of mobility?	46 (31)
Where there is no evidence to differentiate between medications in terms of efficacy, tolerability and side effect profile, and where clinical expertise allows, the medication with lowest cost base should be used.	Primary Laxative (PL): Bisacodyl	5 (3)
	PL: Senna	27 (18)
	PL: Lactulose	3 (2)
	PL: Glycerol	3 (2)
	PL: Docusate	39 (26)
	PL: Sodium Picosulphate	14 (9)
	PL: Macrogols	33 (22.0)
	PL: Other	8 (5.3)
	PL: None administered	14 (9.3)
4.3 The combination of a softening & stimulating laxative is often required. Optimisation of a single laxative is recommended prior to the addition of a second agent.	Was a combination of a softening and a stimulating laxative used?	68 (45)
	^a^Was optimisation of a single laxative achieved prior to the addition of a second agent?	48 (32)
4.4 The laxative dose should be titrated daily or alternate days according to response.	^a^Was the laxative dose titrated: Daily	20 (13)
	^a^Was the laxative dose titrated: On alternate days	11 (7)
Clinical guidelines: Opioid induced constipation		
5.1 The development of OIC should be anticipated. A bowel regimen should be initiated at the commencement of opioid therapy	Was a bowel regimen initiated at the commencement of opioid therapy?	79 (53)
5.2 In the management of OIC optimised monotherapy with a stimulant laxative is essential followed by the addition of a softener if required.	Was optimisation of a stimulant laxative achieved prior to the addition of a softening laxative?	17 (14.2)
Clinical guidelines: Intestinal Obstruction		
6.1 A stool softener should be considered in partial intestinal obstruction (IO). Stimulant laxatives should be avoided.	In patients with partial IO: was the use of a stool softener considered?	8 (50)
	In patients with partial IO: were stimulant laxatives avoided?	1 (8)
6.2 In complete IO, the use of all laxatives should be avoided as even softening laxatives have some peristaltic action.	In patients with complete IO: were all laxatives avoided?	1 (14)

^a^ Not recorded in 15% or more of case-notes

### Constipation assessment

A comprehensive assessment, including taking a full history and performing a physical exam, was recorded for 109 patients (73%). On average 2.29 (SD = 2.00) physical symptoms were recorded, the most frequent including infrequent bowel movements (n = 68; 45.3%), nausea (*n* = 57; 38.0%), abdominal pain (*n* = 50 33.3%), vomiting (*n* = 43; 28.7%), and poor appetite (*n* = 34; 22.7%). At one site patients were statistically more likely to receive a comprehensive assessment than the other two sites (X^2^ (2, *N* = 145) = 6.048, *p* = .049). Age (*p* = .51), gender (*p* = .31), level of independence (*p* = .53), and reason for admission (end of life care compared to physical or psychological support) (*p* = .18) did not significantly impact on the likelihood of receiving a comprehensive assessment.

A lack of standardised documentation for assessment was evident. Data abstractors noted the use of both online and paper notes to document bowel assessment, making it more challenging to access all the relevant information. The Bristol Stool Chart (BSC) [31] was recorded for 144 (96%) patients within 24 h of admission, however, it was unclear if the BSC was used to reassess constipation during their time at the hospice. Two sites reported a “top to toe” assessment for 20 patients (13.3%), suggesting a full physical exam took place during the assessment.

The nurse was involved in 127 (84.7%) of initial assessments across the three sites, however, variability was evident in the involvement of other members of the MDT. At one site, assessments were conducted solely by the nurse (*n* = 28; 56%), doctor (*n* = 7; 14%), or HCA (n = 2; 4%), and 10% (*n* = 5) were conducted by both a doctor and nurse. The second site had 100% (*n* = 50) patients assessed by the doctor, nurse, and HCA. The third site had 39 patients (78%) assessed by both the doctor and nurse, eight patients (16%) assessed solely by the nurse, and two patients (4%) assessed solely by the doctor.

A quarter of case-notes had no recorded information on the relevance or use of digital rectal examination (DRE). Twenty-five case-notes (17%) recorded that a DRE had been performed after three or more days without a bowel movement. Only two case notes (1%) reported completion of a DRE when the patient complained of incomplete evacuation. The use of a DRE was not applicable to 50% of case-notes reviewed (*n* = 75).

### Constipation management

These findings will be focused on the key areas of prevention, non-pharmacological management, and pharmacological management.

#### Prevention

Education on preventative and non-drug measures was documented in 30 (20%) reviewed case-notes, with the majority provided at one site. Participants at one site were significantly more likely to receive education compared to the other two sites (X^2^ (2, *N* = 145) = 69.076, *p* < .001). One site did not provide any documentation in relation to the provision of education on preventative measures. Age (*p* = .70), gender (*p* = .19), and reason for admission (end of life care compared to physical or psychological support) (*p* = .36) did not significantly impact on the likelihood of receiving education. However, patients who did not require assistance with mobility were more likely to receive education on preventative and non-drug measures [X^2^ (3, *N* = 142) = 12.56, *p* = .006] than those requiring assistance or who were bed-bound.

#### Non-Pharmacological management

Non-pharmacological strategies were recorded in 79 patient case-notes (53%). Such strategies included considerations of diet and fluids (*n* = 55; 37%), ensuring privacy (*n* = 52; 35%), mobility (*n* = 46; 31%), and optimised toileting (*n* = 28; 19%). Evidence of non-pharmacological strategies were not explicitly recorded on 41 case-notes (27%), and the remaining 20% of case-notes identified non-pharmacological strategies as not discussed or not applicable. Age (*p* = .55), gender (*p* = .86), level of independence (*p* = .43), and reason for admission (end of life care compared to physical or psychological support) (*p* = .46) did not significantly impact on the likelihood of receiving non-pharmacological intervention. However, there were statistically significant differences between sites (X2 (2, *N* = 142) = 11.962, *p* = .003).

#### Pharmacological management

Laxatives were recorded for 144 (96%) patients. The most frequently administered laxative was Sodium Docusate, a softening laxative, with approximately a quarter of patients 26% (*n* = 39) patients receiving this as their primary laxative and 19% (*n* = 28) receiving it as a secondary laxative. This was followed by Senna, a stimulant, with 18% of patients (*n* = 27) receiving it as a primary laxative and a further 11% (*n* = 17) receiving it as a secondary laxative. Of the patients who required multiple laxatives (*n* = 115), a combination of a softener and a stimulant were used in 59% of cases (*n* = 68). As would be expected, suppositories were more commonly used as a third line of pharmacological management, with Bisacodyl suppositories (n = 17; 11%) and Glycerin suppositories (*n* = 13; 9%) ranking as the top two third level treatments. In a third of patient case-notes (*n* = 49; 33%), no information was recorded regarding the titration of laxatives. Titration of laxatives daily or on alternate days were recorded for 13% (*n* = 20) and 7% (n = 11) of patients respectively. Daily titration varied from 0 to 30% across sites.

### Opioid induced constipation (OIC)

Eighty percent (*n* = 120) of patient notes evidenced receipt of opioids; 17 of whom were on more than three opioids. Patients who were prescribed a higher number of opioids were also prescribed a higher number of laxatives (*p* = .036). Out of the 120 patients receiving opioids, 17 (14.2%) achieved optimisation of a stimulant prior to the addition of a softening laxative. Details on optimisation of a stimulant prior to the addition of a softening laxative was not recorded for 18.3% of patients (*n* = 22).

### Intestinal obstruction

Partial or complete bowel obstruction was not applicable to the majority of patients (*n* = 132). In the 16 patients who had data recorded regarding the use of a stool softener for partial intestinal obstruction, 50% had received a softener (*n* = 8) and 50% had not (*n* = 8). Stimulants were only avoided in one patient with partial obstruction. Seven case-notes recorded complete intestinal obstruction, only one of which showed the avoidance of all laxatives.

## Discussion

This study examined the recorded clinical practices of the assessment and management of constipation for patients with advanced cancer in three SPC inpatient units, and identified variations within and across sites in one organisation in both assessment and management practices from clinical guidelines recommendations.

Approximately three quarters of patients in this study received a comprehensive assessment, and other related symptoms such as nausea and vomiting were also recorded, demonstrating good clinical practice and a holistic approach to assessment. Globally it is advocated in both clinical guidelines and research that the assessment of constipation should combine the following elements: use of the Rome III criteria [13]; a medical history; and a physical exam [14–18]. In the hospice population, great diversity in the physical capacity of individuals (33.3% independent and 21.3% bedbound), calls for assessment to consider not just the patient’s bowel movements, but also a full history of their general health, needs, and circumstances as part of person-centred, holistic palliative care [32]. However, the findings showed no evidence of the use of standardised documentation for assessment or any further follow up, therefore, this may contribute to subjectivity in the assessment process and potentially overlooking patients who are constipated but their symptoms not appropriately documented to help reach a diagnosis.

The guidelines identify best practice as both constipation assessment and management delivered within a MDT with a clearly identified clinical lead and active communication between all team members [18]. Furthermore, a recent meta-synthesis of qualitative evidence identified the key coordinating role of the nurse in palliative care between the patient, family, and other HCPs as a fundamental aspect of their overall role [33]. In line with the guidelines and this meta-synthesis, the nurse had a leading role in the assessment and management of constipation, with variable involvement from other members of the MDT as appropriate and when required. Possible reasons for variability may be attributable to limits in guideline diffusion within the SPC unit, or to perceived role in constipation assessment and management, or due to staffing availability, therefore, additional signposting may support other roles within the SPC setting to identify when their expertise is required across the assessment and management process.

Despite the clinical guideline’s recommendation of the use of DRE, only 25% of patients received a DRE after three days without a bowel evacuation, however, clinical judgment is key to consider when interpreting this finding. Given the clinical pathology of many patients within SPC such as reduced mobility, reduced oral intake, and pharmacology, the cause of constipation can often be identified without an invasive procedure such as a DRE. In addition, almost half (*n* = 65; 43.3%) of the patients included in the chart review were admitted for end of life care. The guidelines state that when caring for a patient who is at the end of life, rectal intervention is rarely necessary [18], therefore, it was appropriate to see limited use of this intervention.

A palliative care approach calls for symptom management to be addressed as a multidimensional experience, considering the non-physical aspects of pain within assessment and management [34]. Pharmacological management was well developed and aligned to the guidelines, which state that the medication with lowest cost base should be used where there is no evidence to differentiate between medications in terms of efficacy, tolerability and side effect profile [18]. A Cochrane review of clinical trials highlighted no evidence to support the efficacy of one laxative over another [20], therefore, laxatives with a lower cost base should be selected. In the UK, Senna, lactulose, and docusate are the three oral laxatives with the lowest cost base (£0.80, £1.30, and £1.95 per 14 day treatment respectively [35]), and these options were used as primary laxatives for approximately half of patients (46.0%), mirroring findings from previous hospice-based research in the UK and USA [10, 21]. However, findings highlighted that pharmacological strategies were more likely to be documented, resulting in a lack of evidence that non-pharmacological interventions were utilised and questioning the use of a holistic approach to care [36]. Best practice is based on a balance between strategies for prevention and self-care and prescribed oral and rectal laxative therapy [19], however, this is not reflected in the current study. Conversations around the prevention of constipation, which were a key element within the guidelines, were only recorded on 20% of patient charts. Whilst equal attention should be paid to optimised toileting, lifestyle modifications, and adjustment of activity levels [18] as well as pharmacological management, non-pharmacological interventions were only documented in 53% of case-notes, compared to 96% documenting pharmacological intervention. These findings call for a greater awareness of the holistic approach necessary in the prevention and management of constipation in palliative care. Whilst patients may be implementing these independently of their hospice plan of care, preventative and non-pharmacological strategies are of equal relevance to pharmacological care and therefore should also be discussed by HCPs and recorded alongside pharmacological intervention.

Previous studies have shown that only a small proportion of the cost of managing constipation in a SPC inpatient unit was related to drug expenditure (13%), compared to 85% of cost for staff time [37]. The cost to older adults can be seen in terms of the reported negative impact on physical, psychological, and social well-being in everyday life [38]. This cost to older adults was also found in a recent systematic review within SPC settings, therefore, it is essential that this symptom is well managed [8]. Good clinical record keeping is an integral component in good professional practice and the delivery of quality healthcare, and would enable enhanced communication within the MDT, reducing the staff time required to manage constipation [22, 23, 39]. Effective assessment and management of constipation through adherence to clinical guidelines along with good documentation of all aspects of assessment and management may assist in minimising the burden to both staff and patients.

 Interpretation of the findings must be undertaken cautiously, due to the inability to differentiate between data that were not recorded, and clinical activities that were not undertaken. Non-recorded data limits the ability to provide a full picture of what happens in this setting regarding the assessment and management of constipation. However, this study is able to provide useful insights into data recording and some ways in which the problem of constipation is it being managed within this practice setting. Strengths of this research are also acknowledged. Through the use of Gilbert’s [25] evaluative criteria, the methodological approach has been conducted in a rigorous manner, in particular the training and follow up verification with data abstractors after collecting five case-notes. This rigorous approach has increased the replicability of the study and reliability of the findings.

## Conclusions

This study provides an insight into the recorded clinical practices of constipation assessment and management in inpatient SPC settings. Despite existing clinical guidelines, clinical records show great variability within and between sites. Whilst comprehensive, holistic assessments were evident for 73% of patients, a lack of standardised documentation or evidence of follow up may result in the identification, diagnosis, and subsequent treatment of constipation being missed for some patients within this setting. The nurse, supported by standardised documentation procedures, plays a key coordinating role in identifying and assessing constipation. Application of pharmacological interventions appeared to adhere to clinical guidelines, but prevention and non-pharmacological interventions requires further attention. Accurate documentation is essential for not only identifying areas of practice that require improvement, but areas of good practice. Further education is needed to equip HCPs with the knowledge and skills to perform not only a full assessment, but also to use appropriate non-pharmacological and preventative strategies. Owing to gaps in recording, it is vital that SPC settings consider ways to support the implementation of new documenting and recording strategies which are readily available to the MDT to enable transparency of care and maintain good clinical standards. Future research should consider identifying staff knowledge of constipation, and the use and effectiveness of non-pharmacological interventions across clinical settings for this group of patients.

## Additional files

Additional file 1: Management of Constipation in Adult Patients Receiving Palliative Care Guidelines Summary. (DOCX 326 kb)

Additional file 2: STROBE Statement—checklist of items that should be included in reports of observational studies. (DOCX 40 kb)

## Data Availability

The datasets used and/or analysed during the current study are available from the corresponding author on reasonable request.

## Abbreviations

BSC: Bristol Stool Chart
DRE: Digital rectal examination
HCPs: Healthcare Professionals
MDT: Multi-disciplinary team
OIC: Opioid induced constipation
ORECNI: Office for Research Ethics Committees Northern Ireland
SPC: Specialist palliative care
UK: United Kingdom

## Footnotes

1 The ADAPTE process provides a systematic approach to adapting guidelines produced in one setting for use in a different cultural and organizational context. The adaptation consists of three main phases; set-up phase, adaptation phase, and finalization phase.
