# Supplementary material for: Examining constipation assessment and management of patients with advanced cancer receiving specialist palliative care: a multi-site retrospective case note review of clinical practice
Source: BMC Palliat Care. 2019 Jul 15;18:57. doi: 10.1186/s12904-019-0436-3 (PMC6631441; doi:10.1186/s12904-019-0436-3)
Supplement: Supplementary file 1 — Management of Constipation in Adult Patients Receiving Palliative Care Guidelines Summary. (DOCX 326 kb) [file 12904_2019_436_MOESM1_ESM.docx]

Supplementary Table: Management of Constipation in Adult Patients Receiving Palliative Care Guidelines Summary

| Recommendation 1: Constipation Assessment  **Key finding** A comprehensive assessment is required to accurately diagnose the presence and potential causes of constipation in patients with life-limiting illnesses. | |
| --- | --- |
| 1.1 | A thorough history and physical examination are recommended as essential components of the assessment process. |
| 1.2 | Constipation assessment scales may be useful in encouraging patient self assessment or when communication is difficult. Due to a lack of evidence in the use of constipation assessment scales in day-to-day clinical practice they are not recommended for routine use. |
| 1.3 | A digital rectal examination (DRE) is required to exclude faecal impaction if it has been more than 3 days since the last bowel movement or if the patient complains of incomplete evacuation (following appropriate DRE training). |
| 1.4 | Caution is advised when considering a DRE in immuno-compromised or thrombocytopaenic patients. |
| 1.5 | A plain film of the abdomen (PFA) is not recommended for routine evaluation but may be useful in combination with history and examination in certain patients. |
| Recommendation 2: Prevention  **Key finding** Preventative measures for constipation should be ongoing throughout the patient’s disease trajectory. | |
| 2.1 | Education on the importance of non-drug measures is essential to enable patients and caregivers to take an active role in constipation prevention. |
| 2.2 | Medications should be reviewed in order to identify potentially constipating agents and prophylactic laxatives prescribed when appropriate. Unless there are existing alterations in bowel patterns (bowel obstruction or diarrhoea) all patients prescribed regular opioids should be started on a laxative regimen and receive education on bowel management. |
| Recommendation 3: Non-Pharmacological Management  **Key finding** Non-pharmacological strategies in the management of constipation are at least as important as the use of pharmacological agents. | |
| 3.1 | Attention should be paid to the provision of optimised toileting while ensuring adequate privacy and dignity for all patients |
| 3.2 | Consideration should be given to lifestyle modification including the adjustment of diet and activity levels within a patient’s limitations |
| Recommendation 4: Pharmacological Management  **Key finding** a. Pharmacological agents are a necessary component of the management of established constipation in life-limiting illness. b. There is a lack of evidence to support the use of any one laxative over another. | |
| 4.1 | 1 The choice of laxative should be guided by individual patient preference and circumstances. |
| 4.2 | Where there is no evidence to differentiate between medications in terms of efficacy, tolerability and side effect profile, and where clinical expertise allows, the medication with lowest cost base should be used. |
| 4.3 | The combination of a softening and a stimulating laxative is often required. Optimisation of a single laxative is recommended prior to the addition of a second agent. The ratio of softener: stimulant should be guided by faecal consistency. |
| 4.4 | The laxative dose should be titrated daily or alternate days according to response |
| Recommendation 5: Opioid induced constipation  **Key finding** Constipation is a common and distressing side effect of opioid therapy | |
| 5.1 | The development of opioid induced constipation should be anticipated. A bowel regimen should be initiated at the commencement of opioid therapy. |
| 5.2 | In the management of opioid induced constipation, optimised monotherapy with a stimulant laxative is essential followed by the addition of a softener if required. The current evidence is too limited to provide evidence-based recommendations for the choice of stimulant laxative and selection should be made on an individual basis. |
| 5.3 | Where there is no evidence to differentiate between medications in terms of efficacy, tolerability and side effect profile, and where clinical expertise allows, the medication with lowest cost base should be used. |
| 5.4 | The use of opioid receptor antagonists under specialist guidance should be considered in patients whose treatment is resistant to conventional laxative therapy |
| Recommendation 6: Intestinal Obstruction  **Key findings** a. If intestinal obstruction is suspected, this should be evaluated by history, examination and appropriate radiological investigations. b. Specialist referral for either surgical or medical management should be considered. | |
| 6.1 | A stool softener should be considered in partial intestinal obstruction. Stimulant laxatives should be avoided. |
| 6.2 | In complete intestinal obstruction, the use of all laxatives should be avoided as even softening laxatives have some peristaltic action. |
| Recommendation 7: Management of constipation in the dying patient  **Key finding** In the last days of life, bowel movements become less frequent as a consequence of proximity to death | |
| 7 | As a patient’s level of consciousness deteriorates, oral laxatives should be discontinued. Rectal intervention is rarely required at this stage. |

Supplementary Form: Data extraction form

**Phase 2 Retrospective purposive reviews of patient case-notes (objective 2)**


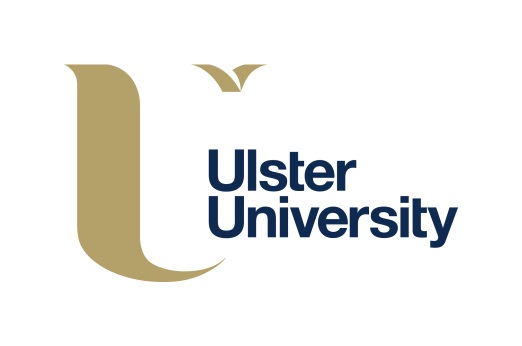


**Data Extraction Form:** **Developing and Evaluating and Educational Intervention for the Management of Constipation (DEMCon)**

| **Name & Role of Person Extracting information** |
| --- |
| **Date of Data Extraction** |
| **Patient Record ID**  The ID should be an anonymous code. **Patient identifiable information should never be recorded and details of the current admission should only be recorded.**  **(NB: Marie Curie Data Extraction Nurse to Log Patient details on separate sheet, to be kept confidential and stored in MC premises in a locked filing cabinet)** |
| **Admission IPU Information**  **Date of admission** ___/______/_________    **Date of discharge** ___/______/_________  **(length of stay in IPU) _____________________**  **Reason for admission**  Symptom Control  Symptom Control + End of Life Care  Respite  Psychological Support  Other (Please Specify) ________________________  **Source of Referral**  GP  Hospital  Nursing home  A&E  Outpatient department  Specialist Palliative Care Team  Generalist Nurse (e.g. District Nurse) |
| **Background Patient Socio-demographic information** 1. Is the patient … Male  Female  2. Patient age ________________years 3. Place of residence Home  Nursing home  Other 4. Family support?5. Level of Independence? Independent  Assistance of One Assistance of Two  Bed-Bound |

| **Clinical Information**  1. Patient primary diagnosis __________________  2. Co-morbidities (list) __________________  3. Date of diagnosis __________________  4. (Current) Hospice Treatment Plan __________________  5. Non-hospice Treatment __________________  6. Medication use (list) __________________ |
| --- |

|  | **Constipation Information**  **Please document reasons for no or N/A in note section** | **Yes** | **No** | **Not applicable** | **Not explicitly recorded** |
| --- | --- | --- | --- | --- | --- |
| **ASSESSMENT** | | | | |  |
| 1. | Did the patient report constipation as a symptom   1. On admission (active) 2. In-patient unit   Notes:  *Please answer all questions from this point forward based on the answer to this question* |  |  |  |  |
| 2. | What symptoms did the patient report?  (Please circle all that apply)  Vomiting  Nausea  Abdominal pain/discomfort  Anorexia  Poor Appetite  Pain on defecation  Rectal pain  Bloating  PR Bleeding  Straining  Gas/Flatus  Infrequent bowel movement  Feeling of incomplete evacuation after bowel movement  Sudden urge to have bowel movement  Hard consistency of stool  Soft consistency of stool  Stool volume/overflow  None  Notes: |  |  |  |  |
| 3. | Was an assessment tool used?   1. If yes, please record assessment tool title? 2. If yes, did this happen within 24 hours of initial contact? 3. What HCP undertook this? (I.e., what disciplines are involved) (Please circle one)   Doctor  Nurse  Other  Notes: |  |  |  |  |
| 4. | What was the duration of time between bowel movements? (Please circle one)  1-3 days  4-7 days  7-10 days  10- 14 days  Other (please specify________________)  Is this a continuous problem for the patient?  Notes: |  |  |  |  |
| 5. | Did assessment include consideration of any of the following components? (Please circle all that apply)  Vomiting  Nausea  Abdominal pain/discomfort  Anorexia  Poor Appetite  Pain on defecation  Rectal pain  Bloating  PR Bleeding  Straining  Gas/Flatus  Infrequent bowel movement  Feeling of incomplete evacuation after bowel movement  Sudden urge to have bowel movement  Hard consistency of stool  Soft consistency of stool  Stool volume/overflow  None  Notes: |  |  |  |  |
| 6. | Was a digital rectal examination (DRE) performed to exclude faecal impaction in the following groups of patients:   - Patients in whom it has been more than 3 days since the last bowel movement? - If the patient complains of incomplete evacuation? - Patients with diarrhea following a period of constipation?   Notes: |  |  |  |  |
| 7. | Was a plain film of abdomen (PFA) performed?  Notes: |  |  |  |  |
| 8. | Did patients with reported constipation have a comprehensive assessment of constipation completed (e.g. PR, palpate abdomen, check for bowel sounds, other)?  Notes: |  |  |  |  |
| **PREVENTION** | | | | |  |
| 9. | Was education on non-drug measures provided in order to enable patients and caregivers to take an active role in constipation prevention?  Was this recorded?  What measures were offered? (Please document i.e., Diet; lifestyle; fluid intake, mobility etc.)  Notes: |  |  |  |  |
| **NON PHARMACOLOGICAL STRATEGIES** | | | | |  |
| 10. | Was there evidence of consideration of non-pharmacological strategies in the constipation management plan? (Please circle all that apply)  Optimised toileting  Privacy  Diet and fluid  Mobility  Other, please list  Notes: |  |  |  |  |
| **PHARMACOLOGICAL MANAGEMENT** | | | | |  |
| 11. | Has the patient been prescribed a laxative?  In patients in whom more than one laxative was used, was a combination of a softening and a stimulating laxative used?  Please circle all that were prescribed:  Bisacodyl suppository  Bisocodyl/ Dulcolax Tablets (stimulant)  Docusate sodium (softening)  Dulcolax Pico Liquid (stimulant)  Enema (Mineral/Vegetable oil)  Enema (Phosphate)  Enema (Microlax)  Glycerin suppository  Lactulose (softening)  Liquid paraffin (softening)  Magnesium hydroxide BP (softening)  Movicol (softening)  Poloxamer & dantron (combination)  Polyethylene glycol (softening)  Senna (stimulant)  Other  Please detail laxative dose and length of time the patient has been administered:  Laxative 1  Dose  Length of time  Laxative 2  Dose  Length of time  Laxative 3  Dose  Length of time |  |  |  |  |
| 12. | Was optimisation of a single laxative achieved prior to the addition of a second agent? (Please detail)  Notes: |  |  |  |  |
| 13. | Was the laxative dose titrated:   1. Daily   Or   1. On alternate days according to response?   Notes: |  |  |  |  |
| **OPIOID INDUCED CONSTIPATION** | | | | |  |
| 14. | Was a bowel regimen initiated at the commencement of opioid therapy?  On admission?  Prior to hospice admission?  If not, was the reason recorded (i.e. loose stools/ incontinent and discharge)  Notes: |  |  |  |  |
| 15. | Was optimisation of a stimulant laxative achieved prior to the addition of a softening laxative?  Notes: |  |  |  |  |
| **INTESTINAL OBSTRUCTION (Consider if Established Diagnosis of Partial/ Complete Obstruction)** | | | | |  |
| 16. | In patients with partial intestinal obstruction:   - Was the use of a stool softener considered?   Notes: |  |  |  |  |
| 17. | In patients with partial intestinal obstruction:   - Were stimulant laxatives avoided? - Was Metoclopramide administered for bowel motility?   Notes: |  |  |  |  |
| 18. | In patients with complete intestinal obstruction, was the use of all laxatives avoided?  Notes: |  |  |  |  |

**Thank you please put this completed data extraction form in a sealed envelope and store in a locked cabinet in MC for collection.**
